# Resolution of low back symptoms after corrective surgery for dropped-head syndrome: a report of two cases

**DOI:** 10.1186/s13104-015-1430-3

**Published:** 2015-10-07

**Authors:** Masao Koda, Takeo Furuya, Taigo Inada, Koshiro Kamiya, Mitsutoshi Ota, Satoshi Maki, Osamu Ikeda, Masaaki Aramomi, Kazuhisa Takahashi, Masashi Yamazaki, Chikato Mannoji

**Affiliations:** Department of Orthopedic Surgery, Chiba University Graduate School of Medicine, 1-8-1 Inohana, Chuo-Ku, Chiba, 260-8670 Japan; Department of Orthopedic Surgery, University of Tsukuba, 1-1-1 Tennodai, Tsukuba, 30585775 Japan; Department of Orthopedic Surgery, Chiba Aoba Municipal Hospital, 1273-2 Aoba-cho, Chuo-Ku, Chiba, 2600852 Japan

**Keywords:** Dropped-head syndrome, Sagittal imbalance, Corrective surgery, Lumbar canal stenosis

## Abstract

**Background:**

Cervical deformity can influence global sagittal balance. We report two cases of severe low back pain and lower extremity radicular pain associated with dropped-head syndrome. Symptoms were relieved by cervical corrective surgery.

**Case presentation:**

Two Japanese women with dropped head syndrome complained of severe low back pain and lower extremity radicular pain on walking. Radiographs showed marked cervical spine kyphosis and lumbar spine hyperlordosis. After cervicothoracic posterior corrective fusion was performed, cervical kyphosis was corrected and lumbar lordosis decreased, and low back pain and leg pain were relieved in both patients.

**Conclusions:**

Cervical deformity can influence global sagittal balance. Marked cervical kyphosis in patients with dropped-head syndrome can induce compensatory thoracolumbar hyperlordosis. Low back symptoms in patients with dropped-head syndrome are attributable to this compensatory lumbar hyperlordosis. Symptoms of lumbar canal stenosis may result from cervical deformity and can be improved with cervical corrective surgery.

**Electronic supplementary material:**

The online version of this article (doi:10.1186/s13104-015-1430-3) contains supplementary material, which is available to authorized users.

## Background

Dropped-head syndrome is defined as apparent weakness of the neck extensor muscles that results in difficulty lifting the head against gravity and consequent impairment of activities of daily living. Its main symptoms include impaired forward vision, neck pain, and myelopathy and/or radiculopathy [1, 2].

We report two cases of severe low back pain and lower extremity radicular pain concomitant with dropped-head syndrome. The patients’ symptoms were relieved after cervical corrective surgery. The present manuscript confirmed to CARE checklist (Additional file 1).

## Case presentation

### Case 1

**Fig. 1:**
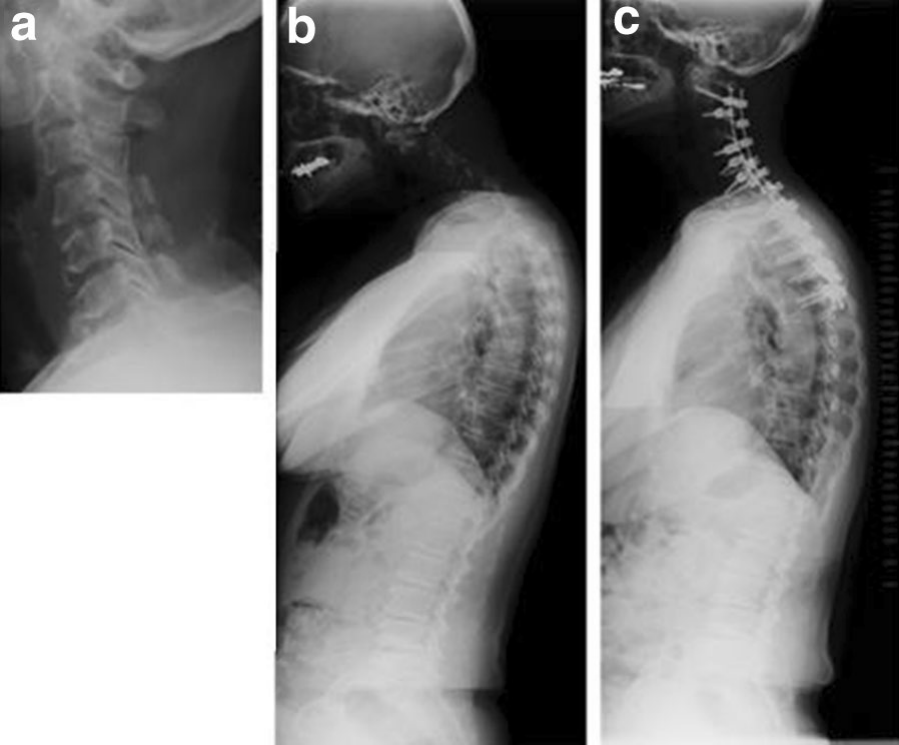
Case 1 radiographs. **a** Lateral plain radiograph of the cervical spine after laminoplasty for cervical spondylotic myelopathy showing normal sagittal alignment. The patient complained of head drop and severe low back and lower extremity pain 2 months after laminoplasty. **b** Lateral whole-spine radiograph showing marked cervical spine kyphosis and lumbar spine hyperlordosis. **c** After cervical corrective fusion (C2–C4), cervical kyphosis and lumbar hyperlordosis, as well as lumbar spine symptoms, were relieved. *C* cervical vertebra

A 72-year-old Japanese woman complained of hand numbness and gait disturbance because of cervical spondylotic myelopathy. The patient underwent 3rd cervical vertebra (C3) to 6th cervical vertebra (C6) laminoplasty (Fig. 1). Her postoperative course was uneventful. Two months after the surgery, she complained of an inability to lift her head because of neck extensor muscle weakness. The patient gradually developed hand numbness. Magnetic resonance imaging (MRI) revealed spinal cord compression both anteriorly and posteriorly at the C4–5 and C5–6 levels. A whole-spine radiograph showed marked kyphosis at the cervical spine and hyperlordosis at the lumbar spine. The angle between C2 and C7 was −35.8°, the 1st thoracic vertebra (T1) slope was 18°, and lumbar lordosis was 43.8° (Fig. 1). The patient complained of difficulty with horizontal gaze, hand numbness, and low back and bilateral leg pain after walking for 10 min.

The patient underwent laminectomy from C3 to C6 and posterior corrective fusion from C2 to T4, which corrected the cervical kyphosis. Postoperatively, the angle between C2 and C7 improved to 17.7°, T1 slope was 16.5°, lumbar lordosis decreased from 43.8° to 31.4°, and the patient experienced relief of her low back pain and bilateral leg pain.

### Case 2

**Fig. 2:**
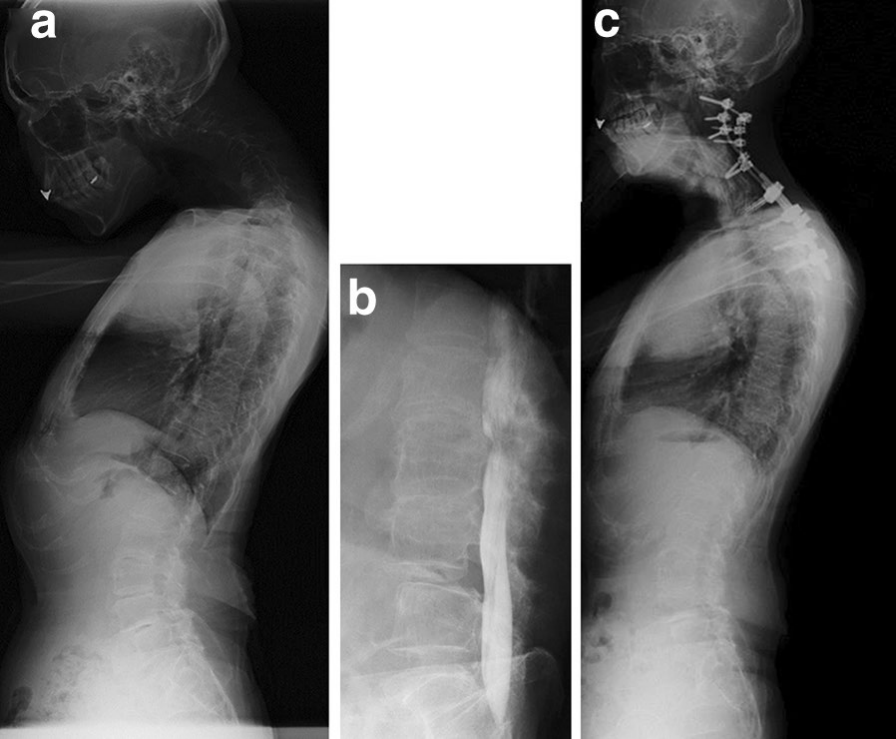
Case 2 pre- and postoperative radiographs. The patient complained of head drop and severe low back and lower extremity pain preoperatively. **a** Lateral whole-spine radiograph showing marked cervical spine kyphosis and lumbar spine hyperlordosis. **b** Preoperative myelogram of lumbar spine showing marked canal stenosis between L2 and L3. **c** Cervical kyphosis and lumbar hyperlordosis were corrected, and low back pain and lower extremity pain were relieved, after cervical corrective fusion (C2–C6). *C* cervical vertebra, *L* lumbar vertebra

A 64-year-old Japanese woman complained of weakness of her left hand, dropped head, and right thigh pain after walking 10 meter. Magnetic resonance imaging revealed anterior and posterior spinal cord compression at the C4–5 and C5–6 levels. A whole-spine radiograph showed marked kyphosis at the cervical spine and hyperlordosis at the lumbar spine. The C2 to C7 ngle was −50°, T1 slope was 17°, and lumbar lordosis was 50° (Fig. 2). Myelography revealed marked lumbar canal stenosis at the L2–3 level. The patient underwent laminectomy from C3 to C6 followed by C4–5 and C5–6 anterior cervical diskectomy and fusion as well as posterior corrective fusion from C2 to T6. Cervical kyphosis was corrected, and the angle between C2 and C7 improved to 22.2°. Postoperative T1 slope was 25°, an increase of 8° from preoperatively. Lumbar lordosis decreased from 50° to 25°. Low back pain and right thigh pain were relieved.

## Discussion

Deformity of the thoracolumbar spine can induce cervical deformity [3]. Smith et al. reported that patients with positive sagittal malalignment tend to compensate with cervical hyperlordosis to maintain horizontal gaze, and that surgical correction of thoracolumbar sagittal malalignment results in resolution of cervical hyperlordosis via reciprocal change. This spontaneous correction of cervical deformity after correction of global sagittal balance by lumbar pedicle subtraction osteotomy has been reported [4].

Conversely, cervical deformity can influence global sagittal balance. The marked cervical kyphosis observed in patients with dropped-head syndrome can induce compensatory thoracolumbar hyperlordosis. The patient in case 2 showed a postoperative increase in T1 slope, suggesting a compensatory extension of the thoracolumbar spine. Low back pain in patients with dropped-head syndrome is attributed to this compensatory mechanism. Extension of the lumbar spine can induce buckling of the yellow ligament and possibly resulting in exacerbation of lumbar canal stenosis and worsening associated symptoms [5]. Therefore, patients with marked cervical kyphosis with compensatory lumbar hyperlordosis experience worsening symptoms of lumbar canal stenosis. If the hyperlordosis and hyperlordosis-related aggravation of lumbar canal stenosis symptoms are actually secondary to cervical kyphosis, low back symptoms can be resolved by correction of cervical kyphosis. In the present cases, compensatory lumbar hyperlordosis was mitigated by correction of cervical kyphosis.

## Conclusion

Lumbar canal stenosis symptoms can result from cervical deformity and can be improved by cervical corrective surgery.

## Consent

Written informed consent was obtained from both patients for publication of this Case Report and any accompanying images. A copy of the written consent is available for review by the Editor-in-Chief of this journal.

## Additional files

10.1186/s13104-015-1430-3 CARE checklist for case reports. The present manuscript confirmed to CARE checklist.
